# Reflections on the human role in AI policy formulations: how do national AI strategies view people?

**DOI:** 10.1007/s44163-022-00019-3

**Published:** 2022-03-03

**Authors:** Henrikki Salo-Pöntinen, Pertti Saariluoma

**Affiliations:** Cognitive Science, Faculty of Information Technology, University of Jyväskylä, Jyväskylä, Finland

**Keywords:** Human-technology interaction (HTI), Human–computer interaction (HCI), AI policy, AI strategies, Human factors, Artificial intelligence, Social transformation, Ethical AI

## Abstract

**Purpose:**

There is no artificial intelligence (AI) without people. People design and develop AI; they modify and use it and they have to reorganize the ways they have carried out tasks in their work and everyday life. National strategies are documents made to describe how different nations foster AI and as human dimensions are such an important aspect of AI, this study sought to investigate major national strategy documents to determine how they view the human role in emerging AI societies.

**Approach:**

Our method for analyzing the strategies was conceptual analysis since the development of technology is embedded with conceptual ideas of humanity, explicit or implicit, and in addition to deepening analysis of explicit argumentation the method enables the deconstruction and reconstruction of meanings and conceptual relations within the strategies, exposing presumptions and tacit commitments of the writers.

**Findings:**

The analysis of the documents illustrates that the general tendency in national strategies is globally dominantly technology-driven as the state of affairs appears to be creating new technologies. However, various human research points such as usability, user experience, sociotechnical and life-based themes are less well represented. Because national strategies are used to develop innovation processes, we argue that future development of national strategies could be improved by taking human research issues more energetically in the agenda.

**Originality:**

Our study elaborates the current trends in AI-policy discourses and discusses reasons and possibilities for more holistic policymaking, making it a valuable resource for policymakers, researchers, and the larger public.

## Introduction

Artificial intelligence (hereafter AI) is increasingly becoming a part of our lives, although it is often an invisible presence. When typing a text, numerous AI programs make the task easier by picking up typos or underlining grammatical errors. Kitchens have invisible apps and other pieces of code which make using stoves, vacuum cleaners, and refrigerators more fluent and more economical to use. Of course, mobile phones and computers with their massive sets of apps are full of AI. Thus, AI is here, and it is increasingly integrated in our everyday life [8, 40, 53, 58, 107].

However, it is not easy to find a clear definition of AI. Definitions vary in that they may concentrate to list functionalities (e.g., adaptability and autonomy) or technological solutions (e.g., machine learning and machine vision) that characterize AI, or they may consider AI as a sociotechnical whole, comprised of the combination of a certain kind of technical artefact and human actions [95, 96]. Here, we rely on Marvin Minsky’s (1967) classical idea of defining AI on the ground of performance capacity [9, 72, 87]. This classic idea was that AI takes care of things which require intelligence from people. One can see that behind this definition is Turing’s (1950) well-known idea that machines can think like people [92, 111], which means that AI applications can perform the same tasks as people, but sameness is defined on the ground of performance capacity rather than on the ground of similarity of processing.

One core measure for the level of AI performance today is its capacity to replace people or to modify the way people have previously worked [87]. For example, autopilots can fly large sections of the routes in which airplanes have normally been operated by human pilots. The routes are operated by autopilots as their performance in normal circumstances was better than humans. Machines do not get exhausted, frustrated, or lose their attention when performing work tasks, even the most mundane ones. Thus, AI can often surpass humans in well-definable tasks, and it is no wonder that massive use of AI shall redefine social work processes [45, 58, 107].

The importance of the growth of AI can be seen in the fact that practically all the major industrial countries have made explicit AI strategies [80]. There is growing scientific literature that has summarized and evaluated some of the strategies, although focusing mainly on their economic and political implications [3, 25, 42]. To analyze possible broader social implications of the strategies, it is important and interesting to pay attention to the underlying intuitive assumptions and tacit commitments. Especially when we think the role governmental working groups give to people in terms of what the strategy papers say about people and their changing life. This is the perspective of our analysis considering the AI strategies of the European Union, Finland, India, France, South-Korea, Germany, Lithuania, Estonia, United Kingdom, Japan, China, and the United States of America.

Indeed, all of technology from a macro perspective concerns how people live, or quality of life [58, 79, 88]. Therefore, it is important to pay attention to the holism of techno-social changes. New technological paradigms have always changed how people live as they modify the ways they obtain their living, form their social relations, and interact with their environment. For example, the steam engine and propeller made it possible to have accurate timetables and consequently, it was possible to reorganize work processes [6, 7, 21].

The paradigms may contribute to the formation of new techno-cultures, which change the entirety of society from habits to laws and ways of living [58, 79]. Agriculture and related technologies gradually replaced nomadic life. People no longer moved from one place to another because surplus made it possible to change social structures and to transit into slave society, social governance changed, and life was renewed. Similarly, the emergence of industrialism transferred people from countryside to cities and changed traditional landowner societies into free democracies [6, 7, 21]. Today, it is essential to think about what an AI-run society will be like.

Much of the AI discussion is performed by people with technical competences [58, 95, 127] as developing AI is understandably an engineering problem [85]. However, one should not think that AI does not essentially change the way people live, and for this reason it is essential to activate social scientists and other human researchers to consider what future life will be like [22]. The contents of national AI strategies have their role in activating this discussion as they include descriptions of required skills for obtaining a wanted AI development [58, 60, 79, 88].

Strategies are documents for what one should do during the next several years. They are primarily used to plan the allocation of resources [11]. Strategies define the goals of national and organizational action and the major actions one must take to reach these goals. Thus, an analysis of national strategies is a way to learn how governmental organizations think and determine what is viewed as important to do and what are the issues of lesser value [58, 60].

It is good to ask what kind of impact the analyzed strategies may have to national policymaking and AI development, and so assess the relevance of studying them. The main direct impacts of the strategies are that they steer national and intergovernmental funding, public procurements, formation of national and intergovernmental innovation ecosystems^1^ and national and intergovernmental legal environments [25, 42, 62, 64]. The analyzed strategies channel funding’s mainly towards educational facilities and research and development activities [25, 42]. To be eligible for funding’s, research groups, innovation ecosystems and educational institutions must comply to funding descriptions, which follow strategic decisions of the public institutions. Through procurements, public institutions have a possibility to create demand for what factors are emphasized in the purchased technologies.^2^ Parallelly initiating, and/or subsidizing innovation ecosystems are actions that reflect social imaginaries and conceptual understandings of the public institutions [58, 59, 111]. Even though the analyzed strategies are not legally binding, they have already generated policy actions over policy cycles (e.g.,[32, 42, 80]; also, see the section considering the national strategy of the United States of America).

It can be argued that strategies in general are high-level plans, and such plans tend to serve only as preliminary guiding thoughts that evolve and change as they are put into action [60, 122, 128]. Therefore, it is very likely that many of the strategies we have analyzed here will not have the concrete influence their writers have hoped for. However, in addition to guiding concrete actions, strategies have the potential of provoking discourses about desirable futures and pathways towards achieving them. This, then again, has impacts on how people perceive technology development and related policy environments [60, 79]. Consequently, from a policy perspective, the strategies we have analyzed fulfil at least a double goal: guide direct policy actions and provide basis for important discourses. For these reasons, analyzing them is of utmost importance.

An important aspect of strategies is their time-span; for example, they examine actions and the world in some 5-year timespan. Thus, commitment to a strategy defines how people will proceed during the next few years in developing AI. The designated timespan is a strength of strategic thinking, but it may also entail risks: if the strategy is mistaken, the consequences may cause harm to resource allocation in AI development processes for a long period. Therefore, it is necessary to consider possible blind spots and gaps in strategies so that they can be discussed promptly. In this paper, the critical questions will be how human dimensions of AI development are understood and what role those dimensions and human research^3^ have been given in the analyzed AI strategies.

Earlier studies have examined the importance of understanding human dimensions related to energy policies to provide more rigorous basis for achieving national implementation goals of green technology [121], and to biotechnology policies to understand why the development of public biobanks faced so much resistance in European countries during the turn of the millennium [58]. In a larger perspective, human dimensions of technology are considered central when developing policies to achieve ethically aligned technology development [22, 58, 79, 88, 98].

This article consists of four chapters: introduction, strategy analysis, discussion on missing questions, and conclusions. The introduction chapter has a subsequent section explaining the methodology of our study, including a definition for what we mean by human dimensions in the context of AI. In addition, the strategy analysis chapter is further divided into two sections considering short descriptions about the role of people in the strategies and an empirical analysis section, in which we provide data of considered human dimensions in a table form.

### Methodology

The aim of our study is to provide information about how human dimensions of AI development are presented in selected AI strategies and reflect how the findings compare to research literature. The produced analysis provides novel understanding about the current state and gaps of AI policy discussions. Therefore, this paper serves as a start for conversation, not as a paper that proposes definite solutions. However, discussions presented here give directions on how coming revisions of AI strategies and their implementations can better incorporate a holistic perspective on AI development.

As the strategies are documents where writers present their ideas in textual form, we used text analysis in the form of conceptual analysis as the method for our study. We chose conceptual analysis as our method because in addition to deepening analysis of explicit argumentation, it also enables the deconstruction and reconstruction of meanings and conceptual relations within the strategies, exposing presumptions and tacit commitments of the writers [57, 86, 99].

Concepts organize the world for us. Therefore, the interest in the meaning and functions of concepts in scientific thinking does not come as a surprise. There are numerous studies devoted to analyzing some key concepts in different areas of science [17, 52, 82, 86, 112]. Conceptual analysis has been used in the context of AI development e.g., to analyze dimensions of governance [13, 37] and ethics [95]. It has also been used to uncover how information systems developers understand humanity [57] and the understanding of users within research areas such as user psychology [91] and human–computer interaction (HCI) [10]. Interestingly, conceptual analysis has also been seen as a method in design research under the term conceptual engineering [16, 26].

Conceptual analysis can mean many different things. Here we are interested in the information contents of concepts. This means that we consider how a concept contributes to the contents of a proposition or a representation [86]. For example, the concept of expert s is different from the concept of medical expert as the latter defines that the person has skills in medicine. This means that in the latter case the concept has the attribute of medicine.

All objects, people and events have theory properties, and the respective concepts have attributes representing those properties. For example, medical doctors are medical experts, because they have medical skills. The analysis of concepts refers to explicating the attributes of concepts. In this way, it is possible to investigate and to analyze the contents of concepts [86].

We are interested in one important issue in the notion of technology. It is common that technology is seen as technical artefacts. Typical examples are electromechanical machines and devices or programs. However, especially in sociotechnical discourses technology refers to the way technical artefacts are used by people in their actions [26, 41, 51, 58].

In this view, technology design can be divided into artefact- and human-technology based. In our analysis we consider what kind of role strategies give to human dimensions of AI development. By human dimensions in technology development, we refer to the roles humans are given in technological development and how the roles are put into action. In the context of technology design and development the two dimensions—description (roles given to humans) and operationalization (how the roles are put into action)—of concepts are equally important, as it has been recognized that abstract definitions (conceptual descriptions) such as ethical principles are not sufficient enough to provide technology or policy developers with the capability of putting the ideas reflected in the abstract definitions into action [39, 57, 73, 75, 95, 104]. In relation to conceptual analysis, description of human roles reflects the information content of the representation of human dimensions and operationalization reflects information content of propositions that are derived from the representation.

The fact that abstract concepts have not been enough for developers of technology and policymakers to put ideas into action is the reason we provide our analysis in two ways: short descriptions of the strategies and a table presenting human dimensions the strategies consider. The table form presents what is mentioned in the strategies (representational level) and the short descriptions provide in-depth analysis of the tacit commitments of the writers, exposing possible contradictions between what is said and how it is perceived to be put into action (propositional level). As an example, the writers of India’s AI strategy say that their high-level goal for AI development is *AI for all*. However, they neglect usability and user experience dimensions of AI development in their strategy, leading to tacitly portraying a top-down view of technology development where people are objects of technology development, not meaningful subjects within the development process. Therefore,—while meritorious in many ways—the strategy falls short in providing actions for achieving *AI for all* from a universal design point of view, which may impede reaching the explicitly stated goals of *AI for all*. As the example shows, our approach provides the reader with an understanding about the ambiguities and complexities that are involved in technology development and the need for considering it from a holistic point of view.

As we reflect the human dimensions provided in the strategy papers to views provided in research literature, we need to define how human dimensions are presented in the used literature. The dimensions can be defined through three large perspectives of human technology interaction (HTI): usability, user experience and sociotechnical aspects. The different views are equally relevant [10, 22] but look at people from different perspectives and therefore hold different problem domains that should be considered in AI development [10, 24, 78]. Usability looks at people as users of technology, which means that the development of technology should take the cognitive functioning of people into consideration for people to be capable to use the developed technology. Compared to usability, user experience largens the scope of considered human dimensions as to involve emotional and motivational aspects of technology use [10, 77, 91]. Sociotechnical aspects of human dimensions can be divided into looking at technology as part of organizational activity—so that technology is perceived as part of social functioning instead of being a separate entity [10, 98]—and to looking at technology as part of the larger non-institutional^4^ social, cultural, and ethical contexts of human lives [10, 58, 81, 91]. The latter description of sociotechnical aspects of human dimensions can also be referred to as life-based approach [88]. The sociotechnical aspects change the role of humans in technology development from users to being reasons for why technology is developed [10, 81, 88, 95].

Through these human dimensions it possible to reflect and discuss AI development as part of desirable societal development [58, 81, 95], understand phenomena such as digital divide and the importance of e-inclusion in the context of AI [58, 81, 123], and perceive novel human-technology interaction (HTI) issues that AI technologies may cause such as appropriate trust [45, 68, 84] or complexities involved in auditing AI systems [63, 127]. Thus, the dimensions provide a multilevel framework for understanding AI design and development in a holistic manner. It is the framework to which we compare how human dimensions are understood within the analyzed strategies.

The nascent field of AI auditing is a practical example of the importance for a holistic view towards AI development. Approaches in the field emphasize a need for interdisciplinary and actionable means for assessing and mitigating unwanted impacts of AI technologies, such as biased results^5^ and loss of privacy. Current approaches consider algorithms [63] or the development processes [127] of AI technology as the main objects for auditing. The point of emphasis does not indicate a dichotomy between the two approaches but points out differences in how recognized ethical and governance principles are considered to be transformed as actionable [95, 108].

Related to AI auditing, it is widely noticed that one core factor for principles to be actionable (and measurable) is that AI technology should be explainable [23, 27, 37, 46, 62, 63]. Explainability^6^ of AI is then again understood as an interrelation between technical solutions [23, 46, 63], action analysis^7^ [45, 54, 62, 90] and analysis of level of use^8^ [37, 63]. Together, they enable the development of different performance measurement and auditing levels so that auditing processes can respect the changing risk and task environments of AI technologies.

All the analyzed strategies consider explainable AI as an important issue to be discussed. However, many of them reduce it as to mean technical transparency and refer to it as the black box issue.^9^ An in-depth comparison between how human dimensions of AI development and explainable AI are perceived in the strategies is a wide question and as such is its own research approach and a good topic for a subsequent article.

#### Sample selection

As the focus of this article is to provide a view of the current state of AI policy discussions from a novel point of view, our selection of AI strategies for analysis was guided by a large enough geographical and cultural coverage.^10^ However, without the pursuit to cover all possible strategies, as that would reduce the possibility to provide in-depth descriptions of the strategies as it requires lots of text space. In addition, we wanted to cover countries with large AI research and development capabilities^11^ such as China and the United States of America to which the intergovernmental strategy of the European Union (EU) can also be considered. We also wanted to see how the EU strategy affects the strategies of its member states, which is why we wanted to cover national strategies of countries from different geographical locations within the EU and different degrees of maturity in relation to AI development and implementation.

We ended our selection process in May 2020. At that time there was no national AI strategies from African, Oceanian, or Southern-American countries and many Asian, European, and North-American countries were missing national strategies. Therefore, our selection process was also guided by the availability of strategies. We acknowledge that the sample countries could be geographically and culturally larger, but as our aim is not specifically to analyze cultural differences within the strategies, but to open a new angle to the discussions of AI strategies and AI policy in general, we perceive that our sample countries are versatile enough to avoid a biased standpoint towards global AI policy discussions.

To avoid interpretations that there are intended value judgements in the ordering of the analyzed national strategies, we cover them in a randomized order. The only exception is the strategy of the EU, which is placed as the first analyzed strategy as it provides a framework for the reader to interpret how the views of the EU level strategy is reflected in the strategies of analyzed EU member states. Our sample strategies are from the European Union, Finland, India, France, South-Korea, Germany, Lithuania, Estonia, United Kingdom, Japan, China, and the United States of America.

## Strategy analysis

The development of technology is embedded with conceptual ideas of humanity, explicit or implicit [57, 58, 60, 79, 118, 119]. This is elaborated in the definition of technology as a combination of technical artefacts and human activity to fulfill defined objectives [41, 88, 100]. It is axiomatic that one needs the right tool to achieve a wanted outcome, but less obvious that the perceived concept of human in the development or choosing of the tool might lead to unwanted or biased results [57].

The working title for this paper was *The Forgotten Human*. After analyzing the strategies further, it became clear that it did not give credit to the intentions of most of the working groups responsible for assembling the strategies, even though it might be illustrative in the case of a few papers. The strategies are by nature focused on clarifying ecosystems required for developing and implementing AI technologies. However, it varies in terms of how relevant the authors of the strategies have regarded defining the relation between humans and AI, or the roles of human at all.

### Short descriptions

The reason for providing short descriptions of each strategy in addition to the table form presentation of empirical data is that the text form descriptions have an in-depth explanatory power. This is necessary for understanding the complexity of the relation of people and technology, and the ambiguity of interpretations provided in the strategies. Whilst x’s in a table hold strong demonstrative power, if left alone, they over-simplify a complex issue by describing very little about the issue itself [58, 93].

#### The European Union

Implementation of the responsible research and innovation (RRI)—initiative to the Horizon-2020 program has made public engagement one strategical emphasis for the European Commission’s (EC) view on AI [33, 120]. Other central concepts for the EU’s AI strategy include responsible AI, trustworthy AI, and human-centered AI [28–31]. All these concepts depict aspects related to the human role in AI development and their influence can be seen in the AI strategies of EU nations.^12^ Thus, understanding the EC’s view on AI is important for understanding the larger context in which EU member states develop their strategies.

The concept of human-centricity is not univocal in the reports describing EU’s strategy towards AI development. In the documents Artificial Intelligence for Europe [28], Coordinated Plan on AI [29] and Building Trust in Human Centric Artificial Intelligence [30], the flourishment of human agency, assurance of human oversight, and ensuring a just work-life transition lay the ground for human-centricity in the context of AI. According to these reports, supporting the flourishment of human agency requires that AI systems empower human beings, allowing them to make informed decisions, pursue their aspirations and help foster their fundamental rights. Human oversight is then again considered to ensure the ethical operation of AI systems. Proper oversight can be achieved through human-in-the-loop, human-on-the-loop, and human-in-command approaches [29].

The emancipatory role of technology is given lesser value—even forgotten—in later EU strategy work. Additionally, the notion of human-centricity is reduced to a synonym for obeying human and basic rights in AI development and deployment [31]. This is contradictory when reflecting on the earlier EU strategy work since obedience to human and basic rights describes the minimum necessities for respecting human dignity but do not function as a holistic approach for defining human flourishment [14, 37].

The White Paper on Artificial Intelligence—A European Approach to Excellence and Trust [31] has gathered ideas from earlier documents related to the EU’s AI-strategy and aims at compiling a comprehensive document for describing the European Union’s strategy. It states that human-centricity and ethical design of AI are core requirements for trustworthy AI development. However, reflecting on the Commission’s earlier work, human-centric AI development should take further steps than just fulfilling “prerequisites” [31 p. 1] for the uptake of AI, such as trustworthiness in the form of legal certainty. Legal certainty is important, but as the Commission’s earlier work emphasizes, human-centric development should aim at fostering the idea of desirable technology and innovations. Otherwise, the concept of human-centricity merely becomes a term in political rhetoric.

As part of supporting the ethical design of AI, the authors of the white paper consider it important to assess social and ecological impacts of developing and deploying AI technology. Additionally, they see strengthening people’s data literacy and basic understanding of how AI works as important steps to empower people and communities to participate in the discussions about what kind of technological development should be pursued for [31]. Moreover, the writers suggest using the AI Assessment List (2019)—made by the EU’s high-level expert group on AI (AI HLEG)—to assess and address social impacts of AI in the development phase and “…transforming the assessment list of the ethical guidelines into an indicative “curriculum” for developers of AI that will be made available as a resource for training institutions” [31 p. 6].

While making a worthwhile proposition of integrating the work of the AI HLEG into concrete strategic actions, by placing social and ethical aspects of technology development as mere check lists^13^ or side courses for developers, the white paper suggests progressing on the demands of technology development. By referencing the AI Assessment List [30], the white paper underlines many aspects that require ex ante deep expertise in issues related to human research and social sciences. This is particularly the case for the AI Assessment Lists’ sections concerning Accessibility and Universality and Social impacts. Therefore, there is a need to have adequate know-how available to understand human dimensions in the process of designing and developing AI-technologies [124, 127].

Considering the emphasis given to ethical design and human-centricity on a conceptual level, the white paper provides a narrow understanding of multidisciplinarity and knowledge management in AI development. By referring to the AI Assessment List, the following problematic understanding of responsibility for knowledge dissemination is also referred to:

*The HR department ensures the right mix of competences and diversity of profiles for developers of AI systems. It ensures that the appropriate level of training is delivered on Trustworthy AI inside the organization* [2 p. 25].

Forming a profile for design teams that ensures a human-centric approach to AI is not only an issue for the HR departments of organizations, but an issue for promoting and developing a systemic understanding of needed skills for the design of trustworthy and desirable AI on the highest political level. From the perspective of ethical and human-centric design, multidisciplinary research and innovation that integrates the points of view of different fields of humanities and social sciences is necessary [88, 124, 127]. Currently, the white paper discusses multidisciplinary research in AI only to illustrate the need for different technical fields to work together [31].

As a conclusion, it would be beneficial for the European Commission to systematize the connection between human-centeredness and ethical design of AI in its strategy work [95]. It would also be consistent that the coming revision of the Coordinated Plan not only “could” but rather should “also address societal and environmental well-being as a key principle for AI” [31 p. 5].

#### Finland

The authors of Finland’s AI strategy Edelläkävijänä tekoälyaikaan (Leading the way into the age of artificial intelligence) [94] understand AI as a largely disruptive technology. Therefore, they see a need for a comprehensive definition of what kind of a role AI should have in society and in relation to humans. The authors define the societal role of AI through the concept of human-centricity.

Human-centricity, then again, is part of the strategy’s 11 key action points, as the 10th action point considers “steering AI development into a trust-based, human-centered direction” [94 pp. 46, 101]. The concept of human-centricity is mentioned in the action point as a precondition for creating an environment of trust. However, the concept is not explicitly defined in any part of the report, and it is used in different ways by referring to a generic adjective, or to the wellbeing of citizens, companies, and society, or to data management that empowers individuals in the data economy era [94].

Despite the concept’s ambiguous use, the report implies that human-centricity will be realized through the national AI program “Aurora AI” and other national AI related initiatives, such as MyData. Aurora AI is a program which aims to sustainably^14^ shift the Finnish public service system to deploy AI in its service providing processes. Its basic idea is that machine learning-based systems use data gathered from individuals to predict their “life-events” [94, p. 85], such as child’s birth, unemployment, etc., and provide the individuals with concentrated information and possible contact details of service providers in a timely manner.

Aurora AI focuses on developing public services, whereas MyData focuses on empowering individual’s agency in the digitalized society all together.^15^ The idea of MyData is to form a data ecosystem where individuals have the right and capability to control all their personal data through a single platform. MyData is therefore an initiative that ensures that Aurora AI together with other digital services are based on people’s consent and aim toward their empowerment, rather than exploitation [83, 94].

On a more general level, it can be said that the writers of the Finnish strategy consider people as a heterogenic group with differing needs considering AI development. This is implied for example in the presented ideas of educational needs related to AI and user engagement. The writers state that the educational systems for lifelong learning must be flexible enough to provide people a chance to choose platforms that suite them best. As for user engagement, the writers emphasize the necessity to understand the variety of backgrounds, interests, and needs people have when considering the process of engaging citizens and stakeholder groups in the development of AI. In addition to acknowledging user needs, the writers perceive wide-ranging engagement as a requirement for decreasing discriminative impacts of AI development and deployment [94].

Despite the incentives to include a variety of perspectives on AI development, the Finnish strategy focuses on individualistic needs of humans. Even talks of inclusiveness refers to inclusiveness of individuals to society. In this way the strategy omits analyzing how communal [79, 108] aspects of human lives are affected by AI development and how the social aspects of human flourishment could be supported through the development and deployment of AI.

#### India

The authors of the Indian National Strategy for Artificial Intelligence [65] perceive AI as a “once-in-a-generation phenomenon” [65 p. 7], which has the power to change people’s lives in such a fundamental way that its outcomes cannot be left to market mechanisms to decide. Therefore, the authors suggest *AI for all* as the national strategy’s main concept*.* This means that the governmental strategy must emphasize development of social good and collaboration between public, private, and academy/research in the development and implementation of AI. Ecosystems must be developed to motivate different stakeholders to work together since multifaceted collaboration is the way to assure that AI benefits the greater good.

Even though the goals of India’s AI strategy—such as development of schooling systems to reduce poverty, production of agricultural innovations through AI to reduce hunger, and improving transport infrastructures to support mobility of people [65]—are well developed to support the use of AI for social good [110], the underlying understanding of AI through technical means may prevent them from being delivered. This is because the target of the application is only one part of what defines a technology’s inclusive dimensions. Others are for example integration of the community’s expectations [120] and taking the dimensions of usability and user experience into consideration in the design of the technology [15, 77]. Otherwise, the process of *AI for all* is run as a top-down process, where people are objects of technology development instead of defining subjects of the process. An example of such incoherence of inclusion can be observed in how the writers perceive low implementation of education technology in India mainly as the result of “unwillingness of teachers and students to adopt technology” [65 p. 35]. There is no mentioning of trying to understand why they are unwilling to use the provided educational technology: it is simply stated as a cause of the users lack in education. This implies an understanding that the usability problems of technology are a fault caused by the user’s capabilities, contrary to taking the capabilities and needs of varying users and usage cultures as the offset for designing technology.

India’s strategy appears in its advantage by being one of the few that considers the need to highlight humans as part of a larger ecological system [65]. However, this concept is not systematically integrated into the strategy since it is narrowed down to mean using AI for reducing the negative ecological impact of humans. A systematic approach would also consider the possible negative ecological impacts of AI technology itself, as for example the development of machine learning systems requires large amounts of energy^16^ [43, 120]. This is another example of how technology can be implicitly considered as a neutral isolated entity to which people and the environment must adjust, contrary to understanding it as part of human activity.

#### France

France’s strategy on AI—AI for Humanity [116]—is one of the most comprehensive national AI strategies, not only in considering technical and infrastructural prerequisites for AI development and deployment but in seeking to answer the question of how meaningful development is achieved. The strategy is based on the document For a Meaningful Artificial Intelligence—towards a French and European Strategy [116] known also as the Villani Report written by a group of AI experts with various academic backgrounds and lead by mathematician Cedric Villani. The name of the strategy underlines its tone; it seeks to redress AI development as a complex systemic process, which should be led by the idea of seeking meaningful progress. The writers of the Villani report explicitly state that AI is not an end in itself and promote the idea of meaningful development as being a result of empowering human well-being whilst producing a competitive national strategy for AI [116].

Human dimensions of AI are incorporated in such concepts as inclusion, human-technology complementarity, impact assessment, ecology, and diversity. Even though dimensions related to each of these concepts are discussed separately within the strategy, they are understood to be intertwined. This is lucid in terms of how the writers emphasize the systemic nature of AI development and deployment [116].

Inclusion of the public is seen to be a prerequisite for developing a democratic society for tomorrow. Inclusion in this context entails including people to discussions considering the use of AI, fostering the skills needed to work and participate in a digital society, supporting fragile segments of population affected by the deployment of AI, and affirming non-alienation of AI-technology by design [116]. The idea of inclusion is also reflected in how the focus areas of AI deployment are chosen on the principle that they serve a general interest of the population. The writers suggest focusing on four sectors—health, environment, transport-mobility, and defense-security—on the notion that in addition to serving a general interest, France has the potential of deploying AI through these sectors [116].

The concept of human-technology complementarity is related to impact assessment of AI on labor markets. The idea of the writers is that by concentrating on the complementary aspects of human-technology interaction, people will not lose their jobs to automation, but new jobs are created instead [116]. The concept is part of a larger construct of promoting ethics by design in the design of applications and education of AI developers.^17^

France’s strategy can be summarized to view e-inclusiveness [34, 123] and meaningful development as emerging from empowering citizens in the age of AI and fostering a diverse view of humanity in the design, development, and deployment of AI. In addition, it calls for a proactive role for government in the pursuance for desirable social change.

#### South Korea

An interdepartmental working group of the government of South Korea released a Mid- to Long-Term Master Plan in Preparation for the Intelligent Information Society as early as 2016. The working group present Realizing a Human-Centered Intelligent Information Society as the main vision for the strategy [71]. They do not explicitly say what they mean by human-centeredness, but it seems to come down to preparing Korean society for societal and economical changes brought about by the large-scale adoption of AI technologies.

The writers of the strategy mention changes in employment structure, growing socioeconomic polarization, and misuse or malfunction of AI to be key societal challenges and threats of the AI-lead fourth industrial revolution. On the other hand, the writers interpret the current industrial revolution to be inevitable and to provide South Korea with the possibility to strive for economic and social wellbeing by becoming a leader in the AI technology development and adoption strategy [71]. This framework is the foundation on which the writers base their policy suggestions.

They suggest a market-lead approach in which the government has a role as a facilitator of a necessary innovation ecosystem in achieving the desired development and as a forerunner in adopting AI technology to governmental practices and public services. Modifying education (including re-education) and social welfare policies is said to be at the core of ensuring that automation of jobs does not lead the socioeconomic polarization of Korean population to grow and in assuring that AI development and deployment is beneficial for all. Combined with initiatives to equalize the opportunities of businesses of different sizes by providing public datasets and key AI technologies to the use of all businesses^18^ and by modifying the judicial landscape to mitigate power concentration to multinational companies that own key software platforms and AI technology solutions—such as Google and Microsoft—the government can help to create new jobs and foster social well-being through the industry-led revolution [71].

The concepts that the authors of South Korea’s strategy use to describe AI technology imply that they are preparing for the emergence of autonomous artificial agents within the concept’s broad meaning (strong AI). They elaborate this by suggesting legislative changes to handle “electronic persons” [71 p. 56] (artificial agents) with judicial responsibilities and rights. In addition, people and technology are referred to as separated agents whose interaction is often described to form a one-way influence from AI technology towards the human. For example, the writers suggest that the roles of humans and ethics should be redefined to fit the age of AI [71], although AI development could also be viewed as a systemic process where ethics and human life are core-defining elements [51, 58, 88, 95].

The absence of human-technology interaction aspects leads the writers to form policy suggestions with an economical and technical focus and thin content on what is required for the technology development itself to supplement human needs and desires.^19^ Therefore, they have laid an objective—human-centered intelligent information society—but with incomplete steps to achieving it. The writers acknowledge the incomplete nature of strategies and suggest compiling a committee to monitor and prevent the negative impacts of AI development and adoption [71].

#### Germany

Human-centered development in Germany’s strategy means reviewing the structural changes that AI will have on work-life and steering the change to a desirable direction. Steering actions include producing a change monitoring system, gathering a consortium to discuss the subject, developing international discourses within ILO and OECD, and increasing research on AI’s effects on the concept of work [36].

The strategy paper draws a systemic view of AI development, but the view is technology-driven. This can be seen in how ethics, work-life, and human-technology interaction aspects are to be implemented ex post facto the design processes of AI systems. They are regarded as relevant only in the implementation phase of technology [36]. It does not have to be so, they could also be included within the design phase [76, 77, 81, 88, 120]. From this perspective, the strategy seems to suggest that other systemic factors of AI development are modified accordingly to fit AI artefacts. The legal framework governing the use of AI technology is considered as an exception in this perspective, as a proper review of needed additions to the legal framework in the context of AI systems is recommended to be done ex ante [36].

#### Lithuania

The strategy paper Lithuanian Artificial Intelligence Strategy—A Vision of The Future [70] outlines how the Lithuanian government will pursue fostering AI development and deployment. The writers of the strategy have pursued to endorse a human-centric approach for AI development in Lithuania. This can be seen in how the strategy is divided into six key sections to guide policy measures related to AI, and the first key section is “Ethical and legal core principles for the development and use of artificial intelligence” [70 pp. 3, 8]. One can see the influence of EU’s policy papers in this section in the use of concepts such as “human-centric” and “trustworthy AI” [70 pp. 5, 8] and the way these concepts are linked to respecting fundamental rights and the technical robustness of AI.

In the aforementioned section of the paper, the writers propose policy measures to establish an AI ethics committee to review impact of AI to fundamental rights and to provide recommendations for the government of Lithuania, create interdisciplinary education in AI for higher education institutions, provide education about ethics of technology in all educational levels, create and foster public engagement measures, and to engage in international regulation and standard setting for AI amongst other proposals [70]. Underlying these proposals is a narrow and vague view of ethics as respecting fundamental rights and applicable regulation and pursuance for technical robustness. Respect for fundamental rights should be viewed as a minimum standard for respecting human dignity. An ethical viewpoint for technology development should seek to comprehend what are the values we should pursue to augment through technology development in addition to fulfilling such minimum prerequisites [14, 38].

The adoption of such a narrow view of human-centered development [88] and ethics is probably one of the factors leading the writers of Lithuania’s strategy to technological determinism in their paper. This can be seen for example in the policy recommendations relating to “National Development of Skills and Competencies Needed for a Future with Artificial Intelligence” [70 p. 14]. The next generations are to prepare “for work with AI” [70 p. 15], the current workforce is “to adapt their workflow to meet the demands of AI” [70 p. 15] and a training program for the general public will amongst other things “communicate the impact that it (AI) will have on the future” [70 p. 16]. However, no one knows the impacts AI will have in societies and our lives in the future^20^—which means the proposed measures try to fit people to presumptions of AI development, rather than asking what kind of development is desired and necessary and what should the role of AI be in constructing the vision.

The writers of the strategy introduce measures such as public engagement, interdisciplinary education on topics related to AI, promoting ethics of technology teaching in all educational levels, and establishing an independent multi-stakeholder AI ethics committee to advise governmental policy, which are important systemic factors in pursuance for desirable AI development and deployment [70]. However, the narrowness of ethical thinking and understanding of human-technology interaction and vague explications of how the topics relate to technology development undermine the good intentions behind the policy proposals.

#### Estonia

The government of Estonia introduced Estonia’s National Artificial Intelligence Strategy 2019–2021 in July 2019. As the headline indicates, the strategy is set for an exceptionally short time-period. This is because an expert group consisting of members from academia, governmental offices, and experts from private sector set to formulate groundwork for the Estonian AI strategy proposed an agile approach for the strategy development process. The expert group presented their views in the Report of Estonia’s Task Force [69] from which the actual strategy was then assembled by the lead of the Ministry of Economic Affairs and Communications.

The idea of the expert group for suggesting an agile approach is to first lay ground for large-scale piloting of AI implementation in the public and private sector during 2019–2021, from which a working group set up to monitor the implementation of the strategy can then gather information to form a long-term AI strategy for Estonia in 2021 [47, 69]. This is one reason why the strategy concentrates on setting basic competences for AI development and implementation in Estonia.

In the context of Estonia’s strategy, basic competences do not mean only technical requirements, but a great deal of attention is given to needed education, requirements for funding, and organizational requirements as well. The expert group has built a comprehensive view of necessary education development. It includes investing in adding basic knowledge of AI to general education, delivering open courses to increase public knowledge of AI, and strengthening multidisciplinary higher education on AI [47, 69].

The set requirements for receiving public funding for AI projects include a sustainability clause. By the sustainability clause, the writers of the strategy suggest that AI solutions—or “kratts”^21^ as the writers call Estonian AI solutions—are to be monitored throughout their life-cycle to make sure they work as intended and do not produce unintended harm. In addition, research projects that aim to understand the complex requirements of implementing AI solutions to different contexts are one of the priority research agendas for 2019–2021, human–robot interaction research being mentioned as one of three key research fields [47].

An important aspect to notice is that the writers of the report state that “The report does not include the topics of adaptation and the social impact related to the implementation of artificial intelligence, as these measures are simultaneously developed by the Ministry of Social Affairs and the Ministry of Education and Research” [69 p. 8]. The same case is with the issue of adapting the labor force to respond to the changes implementation of AI brings forward. It is also noticeable that the expert group’s report includes an overview of ethics related to AI, but that section is not even mentioned in the actual strategy [47, 69]. These remarks make it worthwhile to ask the following question: is the implementation of AI going to be aligned with the needs of societal adaptation and ethical use of AI, if they are separated this way from the actual AI strategy?

#### United Kingdom

The government of the United Kingdom published their strategy for AI Industrial Strategy—Artificial Intelligence Sector Deal as part of a larger industrial strategy in 2018. The strategy is an embodiment of cooperation between government officials, members of industry and members of academia. It followed an independent review Growing the AI industry in the UK [50], from which recommendations were adopted to the final strategy [19, 50].

Unfortunately, regardless of the multistakeholder cooperation, people have been given a minor role in the strategy paper. This can be seen in how the strategy’s common theme is to list technical prerequisites for the successful development and deployment of AI and means to fulfill them. Societal challenges that AI development may raise are not explicitly spoken of, other than the need to assess impacts of automation to different sectors. Otherwise, they are referred to by stating that data ethics is an important factor when deploying AI [19].

The writers set the strategy aim to “create an economy that boosts productivity and earning power throughout the UK” [19 p. 6]. To do so, it is considered necessary that AI is developed in the UK and largely deployed throughout societal sectors. The writers name five foundations which must be noted for the objective to realize, from which “People” [19 pp. 6, 26] is one. On a superficial level, the section concerning people promotes the idea of “Good jobs and earning power for all” [19 p. 6]. When looked at more closely, it focuses on establishing required skills and segmenting populations of interest from the focus point of achieving technical requirements for vast AI development and deployment in the UK. Special attention is given to the need to secure more education on STEM sciences in the schooling system, to retrain people in work-life to be suited for AI and data intensive jobs, to increase higher education in AI, and to attract and retain global high talents in AI [19].

The writers of the Sector Deal consider promoting a “diverse research base” [19 p. 16] in AI and diversity amongst developers of AI as important policy issues. It is left vague what the writers mean by diverse research base, but it is said that it would be beneficial to think of ways of including expertise from other fields^22^ to work in AI. Promoting diversity of developers, on the other hand, is explained to be vital in ensuring that all potential talents are recognized and that the developers represent a realistic view of the demography of the UK [19].

As stated earlier, the Sector Deal promotes the need for cooperation between the government, industry, and academia in AI development. For this reason, a novel council of AI is to be established which consists of experts from these three sectors, and its task is to guide the office of AI in issues related to AI development and deployment [19]. This framework represents a top-down [58, 108, 120] view of societal development. It, together with the Sector Deal’s section concentrating on people,^23^ illustrate how the writers concern the public as an object of AI development and deployment and not as a dynamic participant in the development process.

#### Japan

The Strategic Council for AI Technology introduced Japan’s national Artificial Intelligence Technology Strategy in the form of a report in March 2017. The working group of the Strategic Council explicitly state in the report that the strategy’s road maps for AI development and implementation are “organized based solely on possibilities in terms of technology” [105 p. 5]. Contradictorily, they continue in the same sentence “since it is necessary to resolve issues such as system development, social receptivity, etc. before social implementation, it is possible that more time will be required” [105 p. 5]. To repeat this idea in other words, the writers of the strategy understand the need to resolve other than merely technical issues of AI development, but decide to bypass them in the national AI strategy.

By further analyzing the report, the reader can perceive that the report includes ideas of how to foster the non-technical aspects of AI development. These ideas include active promotion and facilitation of multi-stakeholder open innovation platforms and discussions about AI development and implementation by governmental actors (including dialogue with citizens), taking active part in international standard setting for AI, and augmenting the general public’s knowledge about the possibilities and boundaries for AI [105]. Apart from taking part in international discussions of standards and facilitating multi-stakeholder open innovation platforms, the strategy does not give concrete suggestions or mandate further discussions for these issues.

To clarify objectives for AI development, the writers of the report explicate the “image of society that should be aimed for” [105 p. 5]. As we have stated earlier, images of desirable societies are embedded with presumptions about humanity and the good life [60, 79, 118, 119]. One of the main objectives for AI development is to support hyper-customization of services and goods. This refers to valuing heterogeneity of humanity and/or productivity linked to customization. By examining the Draft AI R&D GUIDELINES for International Discussions [106] produced by the governmental working group of The Conference toward AI Network Society, one can presume that the idea of hyper-customization involves valuing plurality. This is evident in the principle of user assistance, which emphasizes aspects of universal design in the development of AI [106].

The strategy report’s section considering health, medical care, and welfare illuminates presumptions about the relation of humans and technology. Japanese culture(s) is known for containing trends of ideas that do not make clear distinctions between humans and machines [61]. This trend can also be seen in the strategy’s objective of developing medical care towards preventive care, where “body functions can be easily replaced by artificial organs and sensors” [105 p. 7] and in developing welfare to a direction where “General purpose robots are utilized as family members in daily life, solving the problem of nursing care and allowing people to live in peace” [105 p. 7]. These objectives contain projecting human-like cognition as a characteristic of robots without critically evaluating its possible risks [67], or if it is even possible for robots to “understand a person’s intentions” [105 p. 7] and needs.

It is possible that in addition to cultural aspects, the writers of the report do not emphasize the need for critical human-technology interaction research, since nursing robots and longevity of working-aged people have been observed to play a vital part in responding to issues related to Japan’s aging society [56, 105].

#### China

It is worth noticing that the writers of the English version of China’s strategy use the term “integration” to define the desirable relationship between humans and technology. Human-technology integration differs as a concept from human-technology interaction in its way of emphasizing that people and contemporary technology form a symbiotic relationship. This gives the idea of a single functioning unit [97]. The main objective of man–machine “collaboration”, [97 p. 8] or “integration” [97 p. 8] described in the strategy is to enhance the overall intelligence of the symbiotic system and not so much in achieving human-defined objectives.

The writing group of China’s strategy perceives the development and application of AI technology to be the most important factor in achieving economic growth and in increasing social well-being in China. This objective seems to justify placing the studies of social and ethical impacts of AI to support the large-scale application of AI, rather than critically examining it. The writers explicitly acknowledge that the development and adoption of AI technology may have unwanted consequences but point to the paradoxical nature of safe and reliable AI development and adoption [97]; to have reliable AI, one needs a policy framework to control its development and application, but to produce proper policies one needs experience of large-scale application of AI technologies. This phenomenon is also known as the Collingridge dilemma [20].

The paradoxical nature is only partly true since we do need knowledge of technology application to better understand its beneficial nature and risks that it poses. At the same time, technology advancement and application can be, and is, guided by human desires and objectives of what kind of society we want to live in. The difference is that when left undefined, the development is guided by unconscious or implicit objectives. The benefit of explicating the objectives is that they are then placed under public scrutiny, which enhances the discourse of desirable development and acceptability of the developed technology. By clarifying desires and objectives in the strategy, one can enhance their realization and uniformity [44, 59, 76, 81, 120].

However, it seems that the writers of the strategy do not want to place the vision of a desirable society under public scrutiny. This is lucid in how the writers describe the role of the public. Public opinion is not stated to have influence on the design and development of AI, but rather governmental actors are coerced to bring the public to understand the necessity and benefits of AI development and deployment so that the large-scale adoption of AI technology does not phase obstacles caused by public opinion [97]. The interaction between the developers of AI and the public is therefore perceived as non-reciprocal.

The writers talk about people-centered development and producing social wellbeing, but do not clarify what they mean with those concepts and only slightly determine what actions are needed to achieve them in the perspective of AI development [97].^24^ Due to the vagueness of the employed concepts, the determined actions do not have clear goals either. China’s strategy also explicates an objective of gradual improvement of ethical norms, laws, and safety assurance measures according to the five-year cycles between 2020, 2025 and 2030 [97]. This can be considered as leaving them as dimensions that will transform accordingly to the new technological context or as an anticipatory mechanism. Considering the authors’ earlier reference to Collingridge dilemma, the former case is more likely.

#### United States of America

The Executive Order on Maintaining American Leadership in Artificial Intelligence [35] outlines the United States’ national strategy on AI. The strategy establishes six objectives, which are presumed to assure that the United States maintains its leader position in AI development: sustained investment in AI R&D, enhancing access to data, models and computing power, reducing barriers to the use and adoption of AI technologies, producing technical standards to minimize the technology’s vulnerability to attacks, producing an environment of trust in AI technology, training the next generation workforce to be able to take advantage of AI’s potential, and developing and implementing an action plan to protect the advantage of the United States in AI [35].

Although it is mentioned only as one objective in the executive order, research and development (R&D) guidelines form the basis of the United States strategy and play a key role in forming and implementing the action plan to protect the advantage of the United States in AI mentioned in the Executive order [35, 113]. The National Artificial Intelligence Research and Development Strategic Plan: 2019 Update [113]^25^ produced by the White House Select Committee on Artificial Intelligence^26^ goes into more depth in explicating aspects of the AI development than other official policy documents of the government of the United States. Therefore, we examine the United States strategy’s views through The National Artificial Intelligence Research and Development Strategic Plan: 2019 Update.

In the updated AI R&D strategic plan, the select committee concentrates solely on framing the research and development guidelines for AI as guidance for federal agencies of the United States. They set out eight R&D priorities for the strategy: 1. sustaining long term investments in AI research, 2. developing effective methods for human-AI collaboration, 3. understanding and addressing the ethical, legal, and societal implications of AI, 4. ensuring the safety and security of AI systems, 5. developing shared public datasets and environments for AI training and testing, 6. measuring and evaluating AI technologies through standards and benchmarks, 7. providing better understanding of the national AI R&D workforce need, and 8. expanding public and private partnerships to accelerate advances in AI [113].

Viewing the eight priorities of the R&D strategic plan gives a feeling that its writers have a holistic view of AI development and acknowledge people to be at the center of the development. In reality, the strategy is contradictory in how it draws attention to human and social aspects of AI development and deployment. On the one hand, the writers clearly state that aspects of human-technology interaction and social and ethical implications of AI development and deployment are important issues, and that ensuring trustworthy and desirable development for AI requires multidisciplinary approaches including social sciences and humanities. But on the other hand, the strategy has a technically-oriented approach to human-AI interaction, where for example producing ethically aligned AI systems is mostly a technical question of system architecture [113]. This kind of an approach implies a narrow understanding [37, 95, 115] of ethical issues related to technology development and that the ethical and social issues related to Human-AI interaction are already understood and clear to the system developers. However, research demonstrates this is not the case [15, 45, 114].

Another example of the contradictory nature of the strategy is that AI development’s possible impact on work life is mentioned as an addressable social issue in the section considering ethical and social impacts of AI but it is not mentioned in any way in the section considering employment. Rather, the employment section concentrates in questions of how to guarantee a capable workforce that can fulfill the promises of AI development and guarantee that the United States preserves its leading position in the international AI arena [113].^27^

The contradictory character of the strategy is likely due to it being more of a document that seeks to elucidate primary issues in AI development and deployment as largely as possible but does not seek to provide answers or concrete next steps to solving them. This leads to a lack of conceptual and goal uniformity for the strategy.^28^ The writers of the strategy state that the idea behind concentrating on the R&D dimensions of AI development is that governing and regulation proposals will occur as results from the government-supported R&D processes [113]. However, this omits the question about how successful or coordinated can the addressing of the possible proposals be if the strategy does not prepare conceptual or operational ground for it?

### Empirical analysis

National strategies are vital documents as they guide national innovation systems. Such institutional actors as universities, governmental training, education, state supported research activities and technology acquisition decisions are in numerous ways connected to the strategy papers. Strategy papers define what is important and what is worth labor in innovating new technological ideas.

When comparing contents of existent strategies with the major issues being actively analyzed by research communities, it is not difficult to find human issues which would make sense as parts of national strategies. We have identified three multilayered entities of human dimensions that the governmental working groups should consider in their strategies to get prepared for a future where the role of AI technologies is pervasive. We classify the entities as sociotechnical, usability and user experience aspects of AI development [59, 77, 91, 106]. The sociotechnical aspects can be further parsed to consider the description of the pursued society (desirable society) [58, 60, 81, 91, 120], engaging the larger public’s anticipations and desires in the designing processes of AI [58, 81, 91, 120], adaptation of people’s livelihood [5, 12], human rights (HR^29^) impact assessment [4], environmental impact assessment [43, 81, 120], and adaptation of educational systems [79, 91, 108, 124, 127]. In addition, as usability is a wide research area, we identify that usability should be understood as universal design [125] in high-level AI strategies. This is because universal design takes into consideration that people have distinct capabilities and needs as technology users. Therefore, it is an important aspect in inclusive technology design [123, 125].

Due to the ambiguous nature of some of the identified sociotechnical issues, some further clarification is necessary. We consider public engagement as the action of empowering the larger public to participate in the discourse of desirable development as a meaningful actor, as described in the RRI framework [81, 120]. By adaptation needs of labor/livelihood, we mean adaptation needs caused by large scale use of AI technology and there by modification or loss of jobs, not only adaptation needs to ensure competitive AI development [58, 79, 107]. In addition, assessment of environmental impact should include both, the use of AI technology to achieve green growth and assessment of environmental impact of the use of AI technology [18, 43, 81, 110, 120].

Furthermore, we understand the educational system to include basic and higher education as well as continuous learning in work-life. By interdisciplinarity, we mean combination of disciplines from STEM and natural sciences with disciplines from humanities and/or social sciences. This is because we believe expertise from humanities and social sciences are vital for being able to take human dimensions into consideration in AI development [5, 12, 79, 88, 95, 124, 127]. While important, we do not notice the combination of only technical disciplines as an interdisciplinary action in our analysis.

Our method for analyzing the strategies was philosophical text analysis in the form of conceptual analysis. This means, we analyzed the strategies on their explicit and tacit argumentative levels and compared their arguments to the framework of acknowledged human dimensions [86]. From this, we built a matrix (Table 1) presenting how the working groups responsible for assembling the national strategies have considered the aforementioned human dimensions in their final presentations of national strategies. X signifies that the considered subject is taken into account in the respective strategy.

**Table 1:** How human dimensions are considered in the analyzed AI strategies

Strategy	Sociotechnical aspects						Usability	User experience
	Description of desirable society (including guiding principles)	Public engagement	Adaptation of labor/livelihood	Impacts on Human Rights	Assessing environmental impact	Adaptation of educational system (including interdisciplinarity)	Universal design	Promotion of user experience point of views
The European Union	x	x	x	x	x	x	x	x
Finland	x	x	x	x		x		x
India	x		x	x		x		
France	x	x	x	x	x	x	x	x
South Korea	x	x	x	x		x		
Germany	x	x	x	x		x		
Lithuania		x	x			x		
Estonia	x	x				x		
United Kingdom	x		x	x		x		
Japan	x	x					x	x
China			x			x	x	
United States of America	x			x		x	x	x

As can be observed in the short descriptions, conceptual analysis proved as an elaborative method for analyzing the strategies. Human dimensions are abstract constructs which is why one might be able to refer to them on a heading level but miss their vital elements or actions to realize them. Therefore, understanding the strategies on an argumentation level is important. For example, as we consider public engagement as the action of empowering the larger public to participate in the discourse of desirable development as a meaningful actor, we do not count actions of guiding public opinion to match with presupposed outcomes as public engagement. This is why, the national AI strategy of China has been marked as to not have considered public engagement, even though the writers of the strategy have referred to it on a heading level.^30^

Table 1 illustrates how national strategies underestimate the complexity and importance of human dimensions. This emphasis should be changed in the future because technology will change human life so fundamentally.

## Discussion

Human dimensions of AI strategies could be contributed by adding human and social issues directly in the documents.^31^ AI entails the greatest social transformation process since industrialization. Therefore, it is not positive to implement it on a strategical level only by following technical rationalities [44, 48, 58, 74]. Rather, social and emancipatory issues should be central in the agenda of strategy discussions. AI is an important technical innovation, but its most important consequences can be found in the ways it changes our societies and social lives.

Our analysis of a number of important national strategies illustrates that the main effort in developing AI is invested in developing technologies. Strategies focus on technical artefacts and their properties and only a restricted set of issues relevant in transformation of work and leisure processes have been accepted to strategic agendas.

### Missing issues

Missing human issues can be collected under three major HTI-research programs [88]. Firstly, the strategies do not pay essential attention to usability-related themes. These themes would also include ergonomics, human factors and HCI themes. The ultimate question is if people can use technologies. AI is a specific technology, and it may be closed behind the gates of digital divide for many people unless usability issues are taken seriously.

Second, an important human-technology interaction problem is user experience [10, 77, 88]. This can also be called affective ergonomics, emotional usability, or kansei-engineering. The core issue is how people feel and how motivated they are in using intelligent technologies. Emotions are central in human information processing as people decide emotionally the value of other things for themselves. In this regard, they experience new technologies as beautiful or attractive, they can trust or distrust intelligent solutions, and they may also feel competent or frustrated [77, 89, 90]. They can even be hesitant with applying new technologies in complex problems such as autonomous transport. Thus, emotional interaction with emerging intelligent technologies belongs to AI related HTI issues which should not be neglected in any AI strategy.

Finally, AI strategists should pay attention to how technologies should be integrated with human life [58, 79, 88, 95]. This third perspective to human interaction with intelligent technologies is complex and versatile. A technology strategist should ask what important social and human life quality issues are to be improved by means of intelligent technologies, how new technologies should be adapted to the demands of human life, and what will the consequences in society and in human life be when some AI technology is generally adopted. The latter question refers also to ethical and legal regulation of new technologies. It also entails economical and management issues, which are almost globally absent or too narrowly discussed (however, South Korea makes an exception as it calls attention to the economic consequences of adopting AI).

The lucid (possible) social issues related to deploying AI involve but are not exhausted in changes in how we work, including loss of routine work processes through automation, endangering of fundamental human rights, such as privacy and non-discriminatory rights, emerging of new marginal groups socially excluded from society (not able or willing to use emerging technology), and growing complexity of security threats in the form of possible cybersecurity breakages. However, developing technical artefacts as if they have intrinsic value, or seeing their design and development processes as morally neutral, pose less obvious impacts. They lead to developing more and more technology which cause unnecessary demands for people to adapt their needs and anticipations to fit the context of developed technology, and in worst case-scenarios they lead to unnecessary and harmful moral trade-offs.

The development of tracing apps for the fight against the spread of COVID-19 provides a good example of how development and use of AI technology can be involved in unnecessary moral trade-offs. Many proposed and used tracing apps are based on concentrated monitoring of movements and contacts of app users, which has posed the risk of violating the user’s right to privacy. The research consortium of Troncoso et al*.* [109] took the preservation of end user’s privacy as a key principle in the design process of their tracing app, and managed to produce a solution of decentralized monitoring, which included no possibility for human supervision of the data. The solution is called DP3T. This example demonstrates how adding aspects of human and social needs and anticipations ex-ante in the design process of AI will bear outcomes that are more likely to be desirable than assessing technology’s role in the social context or aspects of human-technology interaction ex post facto the design process.

## Conclusions

National strategies are generally rather laconic in discussing human roles in developing intelligent technologies. They are technology driven, but should they be something else? It can firstly be asked why human roles should be opened much more effectively on strategy level and after that what are the main issues national strategies should address? The need for reevaluation is evident in how implicit presumptions of technical progress and attaining the described desirable societies are in conflict in many of the analyzed strategies.

AI like all technologies opens new possibilities to meet the challenges of nature and to organize human living in a new manner. New technical capacities enable people to get their living in a new way and thus live a new kind of life. Technical artefacts are important as they enable people to reach their action goals easier and often make reaching possible [6, 7]. The main justification of any technology is that technology emancipates people.

Technology as emancipator means the capacity of expanding the possibilities of life. Originally, emancipation has referred to freeing one from oppressive social conditions. For example, the rejection of slavery in Rome was an example of emancipation [1, 48, 49, 55, 103]. Life can be restricted if social conditions prevent people from increasing the quality of their life. Many human restrictions are humane i.e., political, and social. However, often the problems of human life have been solved through technical advancements. New ways of treating illnesses require new kinds of technical tools, such as new forms of transportation or new kinds of medical instruments.

The emancipatory role of technology has been one of the main catalysts that have led many individuals and organizations to focus their efforts on creating technologies. Decreasing child mortality, illnesses, hunger, and violence, for example, has been possible with the help of technologies [7, 126]. While child mortality was very high 150 years ago even in developed countries, it started to rapidly decrease at the end of the nineteenth century with improvements in medical understanding, hygiene, and technology [126]. Emancipation in the context of HTI thus refers to the liberation of people by technological means from any circumstances that diminish the quality of their lives.

To better understand the current trends in AI policy discourses and roles people have been given in them, we analyzed 12 AI strategies and examined how human dimensions of AI development are perceived in them. In addition, we asked what role the human dimensions and human research have been given in the strategies. We reflected our analysis on a multilateral human dimensions framework which was derived from research literature. Our method of examination was conceptual analysis and we provided results of our analysis in two ways: through short descriptions of each strategy and a table showing how the analyzed strategies have considered acknowledged human dimensions.

From the short descriptions, one can perceive an important notice about a temporal inconsistency when talking about human dimensions within AI development. By this, we refer to the notice that while many strategies may consider some human dimensions as important factors in AI development, the dimensions are understood to be integrated ex post facto the design of the technical artefacts (see especially the short description of Germany’s AI strategy). This shows that the design phase of technical artefacts is not well enough understood as a possible and vital moment for integrating understanding from human and social sciences to the development of AI technologies. As the example of tracing apps demonstrated, this kind of conception does not reflect reality. Best results come from interdisciplinary design processes that incorporate knowledge from human and social sciences right from the beginning. Otherwise, properties of the technical artefacts set limits for how the perceived human dimensions can be taken into consideration.

Table 1 explicates how some of the acknowledged human dimensions are better integrated into current policy discourses and how some of the dimensions are almost absent in total. For example, describing what kind of societies are pursued through AI development, adaptation of labor/ livelihood, and adaptation of educational systems are dimensions that are considered in almost all the analyzed strategies. Then again, consideration of environmental impact assessment and aspects of usability and user experience are missing from most of the analyzed strategies.

Best way to understand what kind of phenomena our results indicate comes from comparison of the short descriptions and Table 1. Firstly, even though Table 1 shows that adaptation of educational systems is widely considered in the analyzed strategies, the short descriptions show that many of the suggestions of integrating human and social sciences are on unsolid ground—as is in the case of considering them as side courses for engineers, even though the related issues require deep expertise (see for example the short description for the strategy of the European Union). Secondly, the short descriptions provide insights for how the negligence of usability and user experience point of views of AI development—evident in Table 1—undermine well-intended pursuance of social well-being in many of the analyzed strategies. This is a good example of how in the policy discourses technology is implicitly considered as a neutral isolated entity to which people and the environment must adjust, contrary to understanding it as part of human activity.

In addition, when looking at Table 1, the strategies of the European Union and France seem to be comprehensive and consistent. However, from the short descriptions it comes obvious that—while in the case of France’s strategy this holds true—in the case of the European Union’s strategy it does not. Even though the strategy of the European Union is comprehensive, it includes ambiguities in how central concepts describing views on HTI issues, such as human-centric AI, ethical AI, and trustworthy AI are perceived. This appears also as inconsistencies in how the goals of AI development are understood and what action proposals are considered. This observation underlines the complexities involved in technology development and the need for holistic HTI approaches in AI policies. According to our findings, France’s AI strategy can be considered a good benchmark for a holistic AI strategy. Nevertheless, as we mentioned in the methodology section, strategy papers should be considered as representations of thoughts and therefore, it is also important to follow how the policy proposals of the analyzed strategies are put into action.

On the general level, HTI thinking in national strategies is narrow: the AI-strategies discuss of AI in a technical manner and set aside the human role in technology. This is an issue that should be rethought. For example, if the national strategies were used to guide national efforts in AI, the minor role given to people may lead to misguided policies. If people are not important, why should one pay attention to human research skills and knowledge in training new generations of AI designers? Why should designers know about the economy, management, or social information processing, if national strategies do not give any attention to these fields of learning?

The rise of an AI-run society is challenging and will necessarily mean job-losses as intelligent machines can perform tasks which have earlier been conducted by people [40]. However, one should not think that the job losses would necessarily lead to unemployment. One can easily see that the problems of cancer or virology could be solved by having ten times more people working on them than today. The end of some jobs does not mean the end for work life in general [12]. The problem is to find proper ways of organizing new economies so that people can transition from old jobs to new ones.

AI is a new kind of technology that will have holistic effects on our society. Therefore, it would be wise to move in terms of social strategy work from narrow technical thinking to holistic technological thinking which not only concentrates on the development of technical artefacts but would also consider social, and life issues at the same time. Extension of the narrow technical focus would provide better possibilities for eliminating negative consequences and other troubles coming from adopting new technical artefacts into social life [53]. Thus, the recommendation of extending AI strategic thinking from technology to socio-technological discussions is well grounded.

## Data Availability

Not applicable.

## Footnotes

1 A concrete example is creating new governmental positions for coordinating AI policymaking and public–private-academy collaborations related to AI development.

2 However, this possibility is not well enough recognized in public administrations [117].

3 By human research we refer to including social sciences and humanities into the idea of multidisciplinary research and development of AI.

4 Non-institutional means in this context that technology is considered as part of human lives in general and not only as part of work contexts. Therefore, it does not mean that institutions have no effect in this dimension, but that they are not the starting point of examination [10, 58, 91].

5 Bias in this context refers to the phenomena of individual, institutional or social prejudice being transferred to the decision-making processes of AI technology. Such bias may be a result of biased training data of machine learning algorithms, uncritically using decision patterns of people (which inherently involve prejudice) to form decision patterns for algorithms, and/or for under-representation of some group(s) of people in the design and development process of the technology [27, 64, 102].

6 Explainability within AI development is also considered a prerequisite for assessing inherent tradeoffs related to developing ethical AI [45, 54, 62–64, 66] and in enabling human oversight to assure ethical operation of AI as there will always occur situations that have not been predicted in the design phase [15, 45, 64, 68].

7 Action analysis includes analyzing the level of autonomy of the assessed technical artefact.

8 Level of use refers to understanding that different users of AI technology (e.g., operators and engineers responsible for redesign) require a different level of explicability from the technical artefact. As the operator requires understanding of how inputs and outputs relate to one another on the problem domain level, the engineer requires understanding of the technical solutions and reasoning underlying the operating level.

9 Black box is the mainstream way of referring to transparency issues related to AI technology [23]. However, it is not a coherent scientific concept, which is why its interpretation is affected by how AI development is understood in general.

10 We pursued to cover all continents and within the continents we selected geographically disperse countries.

11 Capabilities understood as financial funding and as research studies produced within fields related to AI.

12 The EU has recommended its member states to form national strategies for AI with the idea of ensuring that humans remain in the center of development, deployment and decision making of AI [25, 30].

13 Using checklists for assessing the ethical aspects of technology design has been observed to be insufficient [114]. It is unjustifiable to presume that engineers who have a technical education background should be able to assess multifaceted ethical and social impacts (even if they are provided with a list of the issues) of technology, since it is not their area of expertise [51, 95].

14 Sustainability is understood as acts of engaging stakeholders through-out the lifespans of applications, using AI for public good, providing fair compensations for service providers and following the principles of MyData for empowering service users [83, 94, 101].

15 The developers of MyData perceive such an approach to oppose current understandings, in which people are considered passive objects of the interests of AI-deploying organizations. In addition, they argue that supporting this kind of empowerment of individuals is a requirement for ensuring human rights and trust amongst stakeholders in the era of digital economy and promoting the wellbeing of the whole society [83].

16 Therefore, if this aspect is not considered in the designing phase of the AI ecosystems (as in how the used energy is produced), one might contradictorily increase the negative ecological impact of their actions while pursuing to decrease them by deploying AI.

17 The profile of developers is perceived in an interdisciplinary manner, including expertise from social sciences, ethics, and cognitive sciences in addition to technical disciplines.

18 And additional guidance and support for small and middle-sized companies (SMEs).

19 It has to be mentioned that the writers take possible ripple effects of large-scale adoption of AI technologies into careful consideration and build policy measures to support a humane transformation within the expected industrial revolution [71]. These are important actions, but they concentrate on ripple effects, not to the central part causing the societal reform.

20 This is not to say that foresight mechanisms should not be used, but to emphasize that the assessment mechanisms for evaluating sociotechnical transformation are supposed to be interactive tools for furthering and systematizing discourse of change [76]. They are not to produce deterministic views.

21 Kratt is a creature in Estonian mythology, who is said to be treacherous if left untended [69].

22 The language used in the Sector Deal is an example of how AI may sometimes be referred to being a scientific discipline [85].

23 The section concentrates on framing required technical skills for people to be able to adapt to an AI run society and on establishing ways for luring international talents to work in the United Kingdom.

24 For example, the need for a multidisciplinary approach, including social and behavioral sciences, humanities and law is often underlined in the strategy.

25 The 2019 strategy is an update on the 2016 released National Artificial Intelligence Research and Development Strategic Plan and relies heavily on its statements.

26 The Select Committee on Artificial Intelligence is a subcommittee under the National Science and Technology council.

27 It must be noted that the updated strategy also stresses the importance of international and public, private and academia partnerships to assure trustworthy development of AI. This way the protectionist rhetoric of the writers does not imply a nationally inward focus for the development of AI.

28 The section about safety and security is an example of how trustworthiness, transparency of AI technology and human dimensions of technology design are dealt in a holistic, reciprocal, and consistent way throughout the section [113]. But the holistic approach does not consider the whole strategy.

29 Human rights may refer to the Universal Declaration of Human Rights (UDHR) or to local human rights charters, such as the European Convention of Human Rights (ECHR) or the Cairo Declaration on Human Rights in Islam (CDHRI).

30 This elaborates how conceptual analysis is a good method for detecting and pointing out actions of whitewash.

31 This is for example recognized by the government of Japan. Two years after releasing their first strategy paper, they have introduced a new way of shaping and communicating their strategy in the document AI Strategy 2019—AI for Everyone [56].
